# Comparison of Xpert MTB/RIF Assay and GenoType MTBDR*plus* DNA Probes for Detection of Mutations Associated with Rifampicin Resistance in *Mycobacterium tuberculosis*

**DOI:** 10.1371/journal.pone.0152694

**Published:** 2016-04-07

**Authors:** Arfatur Rahman, Mahfuza Sahrin, Sadia Afrin, Keith Earley, Shahriar Ahmed, S. M. Mazidur Rahman, Sayera Banu

**Affiliations:** 1 International Center for Diarrheal Disease Research, Dhaka, Bangladesh; 2 Oregon Health and Science University, Portland, United States of America; University of Padova, Medical School, ITALY

## Abstract

**Background:**

GeneXpert MTB/RIF (Xpert) and Genotype MTBDR*plus* (DR*plus*) are two World Health Organization (WHO) endorsed probe based molecular drug susceptibility testing (DST) methods for rapid diagnosis of drug resistant tuberculosis. Both methods target the same 81 bp Rifampicin Resistance Determining Region (RRDR) of bacterial RNA polymerase β subunit (*rpoB*) for detection of Rifampicin (RIF) resistance associated mutations using DNA probes. So there is a correspondence of the probes of each other and expected similarity of probe binding.

**Methods:**

We analyzed 92 sputum specimens by Xpert, DR*plus* and LJ proportion method (LJ-DST). We compared molecular DSTs with gold standard LJ-DST. We wanted to see the agreement level of two molecular methods for detection of RIF resistance associated mutations. The 81bp RRDR region of *rpoB* gene of discrepant cases between the two molecular methods was sequenced by Sanger sequencing.

**Results:**

The agreement of Xpert and DR*plus* with LJ-DST for detection of RIF susceptibility was found to be 93.5% and 92.4%, respectively. We also found 92.4% overall agreement of two molecular methods for the detection of RIF susceptibility. A total of 84 out of 92 samples (91.3%) had agreement on the molecular locus of RRDR mutation by DR*plus* and Xpert. Sanger sequencing of 81bp RRDR revealed that Xpert probes detected seven of eight discrepant cases correctly and DR*plus* was erroneous in all the eight cases.

**Conclusion:**

Although the overall concordance with LJ-DST was similar for both Xpert and DR*plus* assay, Xpert demonstrated more accuracy in the detection of RIF susceptibility for discrepant isolates compared with DR*plus*. This observation would be helpful for the improvement of probe based detection of drug resistance associated mutations especially *rpoB* mutation in *M*. *tuberculosis*.

## Introduction

Tuberculosis (TB) remains a major public health problem even after more than 20 years of being declared as a global public health emergency. It is the second leading cause of death among the infectious diseases after Human Immune Deficiency Virus (HIV).The World Health Organization (WHO) estimated 9.6 million new cases and 1.5 million deaths worldwide in 2014 [1]. The emergence of multi-drug resistant TB (MDR-TB) is further complicating the situation and is threatening to jeopardize all the prior gains by global TB control programs in recent years. MDR-TB is defined as resistance to two of the most potent first-line anti-TB drugs, rifampicin (RIF) and isoniazid (INH) with or without resistance to any other first line anti-TB drug [2]. According to a WHO estimate, there were approximately 300,000 new cases of MDR-TB and around 190,000 fatalities from TB worldwide only in 2014[1]. Introduction of newer and rapid diagnostic tools have increased the detection of these MDR/Rifampicin Resistant (RR) cases in recent years. In 2009, only one in every six estimated cases was being detected worldwide whereas in 2013, almost one in every two estimated cases was being detected. Still a big gap remains and approximately 55% of the estimated MDR/RR-TB cases remained undetected worldwide in 2013 emphasizing the need of a wider roll out of rapid detection tools [3].

Rifampicin is a widely used first-line anti-TB drug that works by inhibiting the Mycobacterial ribonucleic acid (RNA) synthesis. The vast majority (around 95 to 98%) of RIF resistance occurs due to mutations in the 81bp (codon 507–533) of the Rifampicin Resistance Determining Region (RRDR) in the gene encoding the beta subunit of RNA polymerase (*rpoB*) [2, 4, 5]. Rifampicin resistance can be used as a surrogate marker for MDR-TB, as more than 90% of RIF-resistant isolates are also resistant to INH, another potent first-line anti-TB drug [4, 6, 7]. Xpert MTB/RIF Assay (Xpert) and Genotype MTBDR*plus* (DR*plus)* are two WHO approved standard molecular diagnostic methods that have been developed for the rapid detection of RIF resistance by scanning the RRDR DNA for associated mutations. These two methods can serve as direct Drug Susceptibility Test (DST) on both smear positive and negative sputum samples [8–10].

Xpert (Cepheid, Sunnyvale, CA) is a proprietary hemi-nested real-time Polymerase Chain Reaction (RT-PCR) based assay which uses five fluorescent wild-type (WT) probes to scan the 81bp region. Studies have shown that all 23 of the most clinically relevant single mutations result in the failure of the WT probes to bind, resulting in a resistant diagnosis by the software [4, 11]. Advantages of this system are its simple and automated, only two hours turn-around time and minimum bio-safety requirements due to its one-vessel system [10–12]. The specificity and sensitivity of Xpert for detection of RIF resistance are more than 98% and 99%, respectively [10].

DR*plus* (Hain life Science, Nehren, Germany), is a multiplex PCR in combination with reverse hybridization based technique that employs 8 WT probes and 4 mutation specific probes (MUT), that target the RRDR [13, 14]. Drop-out of WT probes or hybridyzation of MUT bands indicates RIF resistance, but unlike Xpert, DR*plus* can provide specific information on four of the most prevalent mutations (S531L, H526Y, H526D and D516V). DR*plus* is also highly sensitive (>97%) and specific (>99%) in detection of RIF resistance [9].

Because both the WT Xpert and DR*plus* probes are degenerate, they can detect multiple mutations, and as a result, the specific genotype of the strain cannot be determined. Conversely, the four DR*plus* mutant probes are specific for one mutation, so the genotype of these strains can be known [15]. As both techniques scan the same region of DNA, there is an overlap between the DR*plus* probes and Xpert probes. Xpert probe “A” hybridizes at the same region as DR*plus* probe WT1 and WT2 do. Similarly, four other Xpert probes also correspond to one or more DR*plus* probes [7, 13, 15]. From this agreement of two molecular methods, it is evident that mutation in a particular locus will be detected by delayed or no hybridization of an Xpert probe and lack of corresponding WT band or presence of MUT band in DR*plus*. Our aim was to analyze the agreement level of two molecular DST methods in detection of mutations at RRDR, associated with RIF resistance.

## Materials and Methods

### Sample inclusion criteria

Sputum specimens were collected from the ongoing “*Surveillance of Multi-drug Resistant (MDR) and Extensively Drug Resistant (XDR) Tuberculosis in Bangladesh*” study. This study was approved by the institutional review boards of the International Centre for Diarrhoeal Disease Research, Bangladesh (icddr,b), namely the Research Review Committee (RRC) and the Ethical Review Committee (ERC). A written informed consent was obtained from participants, which was also approved by the RRC and the ERC. Participants who did not give consent were not included in the study. This surveillance study uses a systematic sampling strategy whereby the newly registered pulmonary TB patients are enrolled from 17 different health care facilities across 12 districts of Bangladesh using a site specific sampling interval. The sampling intervals are calculated based on the annual case burden of the relevant facility. The first 260 sputum specimens (collected during the period of September 2011 to July 2013) of this surveillance project were considered in this study. As our aim was to analyze molecular probe hybridization/drop out for the detection of mutation, we decided to include the isolates with RIF resistance detected by any of molecular method in this study. We excluded the isolates which were RIF susceptible in both methods and/or incomplete results in either one (error or invalid in Xpert, TUB band negative in DR*plus*). A total 92 specimens out of 260 fit these criteria and were included in this study. As this study does not aim to compare the overall performance of the two concerned diagnostic tools, exclusion of isolates with error/indeterminate results seemed rational.

### Specimen processing

All sputum specimens (n = 92) were processed by N-acetyl-L-cysteine -Sodium Hydroxide (NALC-NaOH) decontamination method [16]. In this process, 0.5% NALC was used to digest the sputum specimens and to decontaminate, NaOH (4%) and Na-Citrate (2.94%) were used. Equal volume of specimen was added to NALC-NaOH-Na citrate solution in a 50ml centrifuge tube and vortexed for 10–20 seconds. The mixture was kept at room temperature for 15 minutes for decontamination, filled to 45 ml with phosphate buffered saline (PBS, pH = 6.80), mixed well by vortexing and centrifuged at 3000g for 15minutes. Supernatant was decanted carefully and the pellet was re-suspended in 1.0ml PBS.

### Löwenstein-Jensen proportion DST (LJ-DST)

The LJ-DST was performed according to standard protocol [17]. Drug containing media were prepared using 40.0μg/ml of RIF on egg based LJ media. A 1.0 McFarland standard mycobacterial suspension was prepared in sterile distilled water from a freshly grown colony. This standard suspension was serially diluted to four different concentrations (10^−1^ to 10^−4^ cells/ml). Equal volume (10.0μl) of suspension was inoculated to LJ slants with and without drugs, with a platinum loop of 3.0 mm internal diameter calibrated to 10 μl. LJ slants were incubated at 37.0°C temperature. Depending on growth on control media, results were read at 28^th^ day, 35^th^ day and finally at 42^nd^ day. The criterion of resistance was colony growth, equal to or above 1% on drug containing media compared to drug free media.

### Xpert MTB/RIF Assay

Xpert was done according to manufacturer’s instructions [18]. Briefly, Xpert Sample Reagent (SR) was added to unprocessed sputum sample in a 2:1 ratio into a 15ml centrifuge tube and incubated at room temperature for 15 minutes. During incubation period, the samples were mixed by inverting the tubes gently 2 times every 5 minutes. Then 2.0 ml of liquefied sample was transferred to Xpert Cartridge (Ver 3.0) and loaded into GeneXpert IV machine. Within two hours Xpert machine provided the result.

### GenoType MTBDR*plus* Assay

The DR*plus* assay (both Ver 1.0 and Ver 2.0 for 57 and 35 specimens respectively) was done on decontaminated and concentrated sputum specimens. There are three steps- i) DNA extraction from processed sputum specimen, ii) amplification of RRDR region by PCR, and iii) Hybridization of PCR product to the specific oligo-nucleotide probes, immobilized on the strip. All three steps were carried out according to manufacturer’s instructions [15].The DR*plus* strip contains 6 control probes for verification of the test procedures and additional 21 probes for detection of mutation associated with *rpoB*, *katG* and *inhA* gene. The six control probes include a conjugate control (CC), an amplification control (AC), an *M*. *tuberculosis* complex-specific control (TUB), a *rpoB* amplification control, a *katG* amplification control, and an *inhA* amplification control. In order to give a valid result all six control probes must be positive.

### Sequencing of *rpoB* gene and detection of mutation

For sequencing, DNA was extracted directly from sputum specimens. RRDR region was amplified by using *rpoB* specific primers (Forward primer: 5’-CTTGCACGAGGGTCAGACCA-3’, Reverse primer: 5’-ATCTCGTCGCTAACCACGCC-3’). Product size (543bp) was confirmed by agarose (1.5%) gel electrophoresis. PCR product was purified by QIAGEN MinElute PCR Purification Kit. DNA quantification and purification was confirmed by NanoDrop 2000 spectrophotometer. Purified PCR products were sequenced by Sanger sequencing at the Center of Excellence, University of Dhaka, Bangladesh. Sequenced RRDR region was compared with wild type (H37Rv) *rpoB* core region using Nucleotide Blast, online tool of National Center for Biotechnology Information (NCBI).

### Quality Control

For the purpose of quality control, a RIF susceptible isolate (H37Rv, from American Type Culture Collection, ATCC) and a laboratory defined RIF resistant isolate (SB256) was tested by three different types of DST methods (Xpert, DR*plus* and LJ-DST). SB256 was confirmed resistant to STR, INH, RIF and EMB by LJ-DST and Sanger sequencing earlier. H37Rv was properly detected sensitive and SB256 was detected resistant to RIF by all three methods.

### Statistical analysis

Kappa value was calculated for determining concordance of Xpert and DR*plus* with Sanger sequencing data using SPSS Statistics version 17.0.

## Results

### Comparison between molecular and phenotypic drug susceptibility testing methods for detection of RIF resistance

Three different types of DSTs discussed above were performed on all 92 specimens (Individual data points are provided in the supporting information file, S1 Dataset). Of 92 specimens 85 were RIF resistant in LJ-DST and the remaining 7 were sensitive. In Xpert and DR*plus* respectively, 91 and 86 specimens were RIF resistant and the rests were sensitive (Table 1).

**Table 1:** Comparison of molecular and phenotypic DST methods for detection of RIF susceptibility, n = 92.

Molecular DST methods		LJ-DST		Agreement (%)
		S(n)	R(n)	
DR*plus*	S(n)	3	3	92.4
	R(n)	4	82	
Xpert	S(n)	1	0	93.5
	R(n)	6	85	

Xpert and DR*plus* result of all isolates (n = 92) were compared with LJ-DST. The overall concordance of LJ-DST with Xpert and DR*plus* was 93.5% and 92.4% respectively (Table 1). By LJ-DST, respectively 85 and 7 isolates were resistant and susceptible to RIF. Xpert and DR*plus* agreed with LJ-DST at a rate of 100% (85) and 96.5% (82) in resistant cases, whereas in susceptible case the agreement was only 14% (1) and 43% (3) cases respectively. There were 6 discrepant isolates, which were sensitive in LJ-DST but resistant in Xpert.

Again 7 isolates had discrepant result between LJ-DST and DR*plus*, among which 4 isolates were sensitive in LJ-DST but resistant in DR*plus*, and remaining 3 isolates were resistant in LJ-DST but sensitive in DR*plus* (Table 1).

### Comparison of molecular drug susceptibility testing (DST) methods and their probe hybridization pattern

Two molecular DST methods were compared for RIF susceptibility. The agreement between these two molecular methods was 92.4% (Table 2). Among 92 specimens, 91 was detected as RIF resistant and 1 was sensitive by Xpert. DR*plus* detected 85 of 91 specimens as RIF resistant and rest 6 cases were discrepant among two methods. DR*plus* detected 1 specimen as RIF resistant but was found sensitive by Xpert.

**Table 2:** Comparison of Xpert and DR*plus* assay for detection of RIF susceptibility, n = 92.

Molecular DST method		Xpert		Agreement (%)
		S(n)	R(n)	
DR*plus*	S(n)	0	6	92.4
	R(n)	1	85	

Xpert and DR*plus* showed a similar pattern of probe failure. In Xpert, 91 samples were detected to be RIF resistant. Of these, 2 failed probe A, 14 failed probe B, 3 failed probe C, 13 failed probe D, and 59 failed probe E, resulting in probe failure rates of 2.2%,15.2%, 3.3%, 14.1%, and 64.1% respectively (Table 3). There were no samples that failed multiple probes, so all samples were mutually exclusive to one another.

**Table 3:** Probe hybridization pattern of two molecular DST methods.

Xpert probe failure	No of cases (%)	DR*plus* probe (WT failure/MUT binding)	No of cases (%)
A	2 (2.2)	WT2	2 (2.2)
B	14 (15.2)	WT 2/3, WT 3/4, MUT 1	11 (12.0)
C	3 (3.3)	WT 4/5	1 (1.1)
D	13 (14.1)	WT 5/6	1 (1.1)
E	59 (64.1)	WT 6/7, MUT 2 (A/B)	13 (14.1)
None	1 (1.1)	WT 7/8, MUT3	58 (63.0)
		None	6 (6.5)
**Total**	**92 (100)**	**Total**	**92 (100)**

RIF resistance was detected in 86 (93.5%) of 92 samples by DR*plus*. Of the 86 isolates, 2 had mutation in WT2;11 in WT 2/3, 3/4 or MUT1;1 in WT 4/5, 1 in WT 5/6;13 in WT7/ MUT 2; and 58 in WT8/MUT3 probe specific region, corresponding to rates of approximately 2.2%, 12.0%,1.1%,1.1%, 14.1%, and 63.0% respectively (Table 3).

### Probe hybridization pattern and sequence analysis of discrepant isolates

Eight discrepant isolates showed different patterns of probe failure in Xpert and DR*plus* (Table 4). Of them, three isolates failed Probe B in Xpert but two of these were found to be sensitive by DR*plus* and one was MUT3 probe positive. One isolate failed Probe C and three failed Probe E in Xpert but these four were found to be sensitive by DR*plus*. In case of the last discrepant specimen, it was detected sensitive in Xpert but DR*plus* showed it to be resistant with the MUT3 positive. We observed the treatment history of these discrepant cases and it was found that 7 were re-treatment and 1 was new TB case (S1 Dataset).

**Table 4:** Drug susceptibility result of 8 discrepant cases between two molecular DST methods.

Discrepant Cases	DR*plus* Result			Xpert Result	
	WT missing	MUT present	Overall Result	Failed Probe	Result
**D1**	No	No	S	B	R
**D2**	No	No	S	E	R
**D3**	No	No	S	E	R
**D4**	WT8	MUT3	R	B	R
**D5**	No	No	S	B	R
**D6**	No	No	S	C	R
**D7**	WT8	MUT3	R	No	S
**D8**	No	No	S	E	R

R = Resistant, S = Sensitive.

We sequenced the RRDR region for these 8 discrepant isolates. Seven out of eight discrepant isolates (except D4) showed agreement with Xpert and sequencing data (Kappa = 0.6). Two isolates (D1 and D5) that failed in Probe B of Xpert, showed mutation in corresponding position of 81bp RRDR region (del517 and D516Y) in sequencing. Similar concordance was found in case of the isolates that failed Probe C and E. Mutation was found at location 522 (S522P) for the isolate (D6) that failed Probe C. Mutations were found in three different locations 530 (L530L, CTG-CTA)(D8),531 (S531L) (D3), and 533 (L533 P)(D5) of RRDR including one silent mutation. These six isolates were found to be sensitive in DR*plus*. Another discrepant isolate (D7) showed MUT3 positive in DR*plus* but was found to be sensitive in Xpert, sequencing result showed there was no mutation in RRDR region. No mutation was found by sequencing in an isolate (D4) that showed different results in two different methods (probe B failure but MUT3 positive) (Table 5).

**Table 5:** Aligned sequences of discrepant cases (n = 8) and associated mutations.

Codon	507	508	509	510	511	512	513	514	515	516	517	518	519	520	521	522	523	524	525	526	527	528	529	530	531	532	533	Mutation
H37Rv	GGC	ACC	AGC	CAG	CTG	AGC	CAA	TTC	ATG	GAC	CAG	AAC	AAC	CCG	CTG	TCG	GGG	TTG	ACC	CAC	AAG	CGC	CGA	CTG	TCG	GCG	CTG	
D1	GGC	ACC	AGC	CAG	CTG	AGC	CAA	TTC	ATG	GAC	-----	AAC	AAC	CCG	CTG	TCG	GGG	TTG	ACC	CAC	AAG	CGC	CGA	CTG	TCG	GCG	CTG	517del
D2	GGC	ACC	AGC	CAG	CTG	AGC	CAA	TTC	ATG	GAC	CAG	AAC	AAC	CCG	CTG	TCG	GGG	TTG	ACC	CAC	AAG	CGC	CGA	CTG	TCG	GCG	**CCG**	L533P
D3	GGC	ACC	AGC	CAG	CTG	AGC	CAA	TTC	ATG	GAC	CAG	AAC	AAC	CCG	CTG	TCG	GGG	TTG	ACC	CAC	AAG	CGC	CGA	CTG	**TTG**	GCG	CTG	S531L
D4	GGC	ACC	AGC	CAG	CTG	AGC	CAA	TTC	ATG	GAC	CAG	AAC	AAC	CCG	CTG	TCG	GGG	TTG	ACC	CAC	AAG	CGC	CGA	CTG	TCG	GCG	CTG	WT
D5	GGC	ACC	AGC	CAG	CTG	AGC	CAA	TTC	ATG	**TAC**	CAG	AAC	AAC	CCG	CTG	TCG	GGG	TTG	ACC	CAC	AAG	CGC	CGA	CTG	TCG	GCG	CTG	D516Y
D6	GGC	ACC	AGC	CAG	CTG	AGC	CAA	TTC	ATG	GAC	CAG	AAC	AAC	CCG	CTG	**CCG**	GGG	TTG	ACC	CAC	AAG	CGC	CGA	CTG	TCG	GCG	CTG	S522P
D7	GGC	ACC	AGC	CAG	CTG	AGC	CAA	TTC	ATG	GAC	CAG	AAC	AAC	CCG	CTG	TCG	GGG	TTG	ACC	CAC	AAG	CGC	CGA	CTG	TCG	GCG	CTG	WT
D8	GGC	ACC	AGC	CAG	CTG	AGC	CAA	TTC	ATG	GAC	CAG	AAC	AAC	CCG	CTG	TCG	GGG	TTG	ACC	CAC	AAG	CGC	CGA	**CTA**	TCG	GCG	CTG	L530L

Note: Sequences were compared with standard H37Rv 81bp RRDR sequence. Shaded codons in discrepant cases are the mutations and amino acid change is shown at the right side of each aligned sequence.

## Discussion

In this study, the two molecular DST methods showed good agreement among themselves. Both of them showed high level of agreement with the phenotypic LJ-DST. Among the specimens that showed discrepant results between molecular DSTs, Xpert showed higher agreement (Kappa = 0.60) with Sanger Sequencing of 81 bp RRDR core region than DR*plus* (Kappa = 0).

Xpert showed 93.5% agreement with the LJ-DST in detection of RIF resistance and the rate was slightly higher than DR*plus* (92.3%). Discrepancies among LJ-DST and two molecular DSTs are due to detection of more resistant cases by molecular methods which may happen due to two reasons. Firstly, these two molecular methods can detect few silent mutations, which do not affect the phenotypic DST [19, 20]. Secondly, Xpert is a quantitative method, which predicts resistance based on ΔCt value; so it is possible to get variable result than any qualitative method like LJ-DST.

Frequency of Xpert probe failure and frequency of corresponding WT probe absence or presence of MUT probes in DR*plus* have shown a likely pattern, which indicates that there is a sound molecular level agreement in detection of different mutations by the two methods. Overall, we found 92.4% agreement between Xpert and DR*plus* in detection of RIF susceptibility but the agreement in specific mutation detection at molecular level was found to be 91.3%. Another study from Zimbabwe also showed similar level of overall agreement among these two molecular methods though they haven’t shown the agreement at molecular level [21]. Most of the discrepancies found in our study were due to detection of sensitive by DR*plus* but resistant by Xpert. Also discrepancies occurred due to mutations being detected in different location of same isolate, by the two methods. The mutation at codon 531 is most frequent, which varies from 20 to 71% [14, 22, 23]. This region is covered by Probe E in Xpert and WT8/MUT3 in DR*plus*. The frequency of Probe E failure was 64% and WT8 missing/MUT presence is 63% which ultimately reflected the mutation rate in this region. The mutations at codon 526 and 516 are also common which varies from 0–30% in RIF resistant cases as observed in different studies [22–24]. In this study the frequency of probe failure/mutant probe binding at these regions varied from 12–15%. All these findings are also consistent with other studies on South-Asian region as well as other countries [22, 24–28].

Sequencing of 81bp RRDR core region for eight discrepant isolates revealed that, Xpert performed more accurately than DR*plus* assay in detection of mutations associated to RIF resistance. Six mutations identified by sequencing of *rpoB* RRDR are also recognized by Xpert through failure of these six location specific probes but DR*plus* is errant in these cases. This happened as DR*plus* failed to detect three mutations (L530L/ CTG 530 CTA, S531L, L 533 P) occurred in location 530–533, two (517del and D516Y) in 513–519 region and final one in S522P. On the contrary, in two other cases where two mutations (WT8 missing/MUT3 present) were detected by DR*plus* but Sanger sequencing revealed that there were no mutation. Though the exact mechanism of the observed discrepancy is not well understood, but we can assume that, this might be caused by mixed bacterial population (heteroresistant) in the sputum. It is known that, re-treatment TB cases contain higher proportion of mixed type bacilli than primary TB cases [29]. We looked at the treatment history of the discrepant cases and found that 7 of the 8 discrepant cases were re-treatment cases (S1 Dataset). Studies have shown that heteroresistant population in the sputum specimen may cause mixed banding pattern in DR*plus* [30]. Although we did not find any heteroresistant banding pattern for RIF, three of eight discrepant cases were found INH heteroresistant. So, there might be a good chance of mixed strains in the sputum specimens of discrepant cases. Sequencing electropherogram can also provide important information about mixed infection as demonstrated previously [30].Therefore, we investigated the sequence electropherogram for 8 discrepant cases. However, for only one case (D4) we found that both wild (GAC) and mutant (GTC) type peaks were present at codon 516, where the wild type peaks were prominent. This indicated the presence of possible mixed type of bacteria in the sputum. Another explanation for more mutation detection by Xpert, would be the use of molecular beacon probes. Molecular beacon probes are highly specific to its target sequence than any other type of hybridization probes [31]. A study on RIF mono-resistant cases, has showed good correlation of DR*plus* than Xpert, with Sanger sequencing [32]. But our findings contradict that in case of MDR-TB.

From sequencing data we found that there was a silent mutation (CTG 530 CTA) in an isolate but Xpert detected it as a resistant. This isolate is also RIF sensitive in LJ-DST. A study from Haiti also showed a silent mutation that was sensitive in LJ proportion method [33]. Many other studies from different parts of the world showed silent mutations across 81bp RRDR region [22, 34–36]. From this point of view, we can say that, in some cases Xpert probes can not differentiate silent mutations which may cause misinterpretation of RIF susceptibility.

The main limitation of our study is the relatively small sample size and higher proportion of RIF resistant cases. As our main objective was to compare the performance of the two molecular DSTs in detection of RIF resistance, we feel that selection of mostly RIF resistant cases was logical. Though we analyzed a small number of discrepant cases, these results provide an important insight into the performance of molecular methods for RIF resistance detection.

In conclusion, the overall concordance with LJ-DST was similar for both Xpert and DR*plus* assay, but Xpert demonstrated better performance than DR*plus* in detection of RIF susceptibility for discrepant isolates. Overall, the findings of this study could be helpful for the perfection of probe based detection of drug resistance associated mutations especially *rpoB* mutation in *M*. *tubeculosis*. Xpert has a number of other advantages over DR*plus* too. Xpert is relatively easier to perform, bio-safety requirement is minimum, less hands-on time ensures lower probability of human errors and turn-around time is faster. Considering these facts and the better performance of Xpert found in our study, we recommend that the national programs in high TB and MDR TB burden countries may consider using Xpert to screen for MDR TB. As Xpert is being rolled out in different countries, there is scope of further research with larger sample sizes and such research work should be conducted to inform the rest of world about the feasibility of wide scale roll out of Xpert.

## Supporting Information

S1 Dataset Individual Data Points. (XLSX)
